# Bridging the Telehealth Digital Divide With Collegiate Navigators: Mixed Methods Evaluation Study of a Service-Learning Health Disparities Course

**DOI:** 10.2196/57077

**Published:** 2024-10-01

**Authors:** Zakaria Nadeem Doueiri, Rika Bajra, Malathi Srinivasan, Erika Schillinger, Nancy Cuan

**Affiliations:** 1 Department of Epidemiology and Population Health Stanford University School of Medicine Palo Alto, CA United States; 2 Division of Primary Care and Population Health Stanford University School of Medicine Palo Alto, CA United States

**Keywords:** service learning, medical education, access to care, telehealth, telemedicine, health disparities, social determinants of health, digital literacy, vulnerable populations, community engagement, value-added medical education, digital health, digital divide, health equity, collegiate navigator, experimental, education, student, qualitative analysis, technology, mobile phone

## Abstract

**Background:**

Limited digital literacy is a barrier for vulnerable patients accessing health care.

**Objective:**

The Stanford Technology Access Resource Team (START), a service-learning course created to bridge the telehealth digital divide, trained undergraduate and graduate students to provide hands-on patient support to improve access to electronic medical records (EMRs) and video visits while learning about social determinants of health.

**Methods:**

START students reached out to 1185 patients (n=711, 60% from primary care clinics of a large academic medical center and n=474, 40% from a federally qualified health center). Registries consisted of patients without an EMR account (at primary care clinics) or patients with a scheduled telehealth visit (at a federally qualified health center). Patient outcomes were evaluated by successful EMR enrollments and video visit setups. Student outcomes were assessed by reflections coded for thematic content.

**Results:**

Over 6 academic quarters, 57 students reached out to 1185 registry patients. Of the 229 patients contacted, 141 desired technical support. START students successfully established EMR accounts and set up video visits for 78.7% (111/141) of patients. After program completion, we reached out to 13.5% (19/141) of patients to collect perspectives on program utility. The majority (18/19, 94.7%) reported that START students were helpful, and 73.7% (14/19) reported that they had successfully connected with their health care provider in a digital visit. Inability to establish access included a lack of Wi-Fi or device access, the absence of an interpreter, and a disability that precluded the use of video visits. Qualitative analysis of student reflections showed an impact on future career goals and improved awareness of health disparities of technology access.

**Conclusions:**

Of the patients who desired telehealth access, START improved access for 78.7% (111/141) of patients. Students found that START broadened their understanding of health disparities and social determinants of health and influenced their future career goals.

## Introduction

Telehealth is an emergent tool to connect patients with their health care providers [1]. During the COVID-19 pandemic, the number of video visits increased by nearly 50% in the first quarter of 2020 compared to that of 2019 [2]. During this time frame, there was a 63-fold increase in telehealth visits among Medicare patients [3]. Telehealth use in the United States continued to rise, with nearly 38 times as many patients using it in 2021 compared to prior to the onset of the COVID-19 pandemic in 2019 [2,4]. Ensuring that patients have access to their electronic medical records (EMRs) is important for communication and coordination with their health care team and to promote patient self-advocacy [5]. While access to video visits and EMR accounts positively impacts access to health care, fewer than half of adults aged 65 years and older own a smartphone or have reliable internet access in the United States [6]. Additionally, racial minority individuals and patients with low socioeconomic status are less likely to have access to essential devices and high-speed internet and face increased obstacles to digital health literacy [7-10]. Consequently, while telehealth can be a conduit to more accessible health care, many at-risk patients experience heightened challenges in connecting with their providers remotely, which may magnify health disparities [6,7].

In response to shelter-in-place orders during the COVID-19 pandemic, medical practices strove to expand telemedicine options and increase resources to aid patients with telehealth [11]. In an effort to streamline this process, several clinics created video tutorials and step-by-step telehealth training guides for patients [12]. However, these static resources quickly proved to be insufficient, and the need for additional assistance from support personnel became evident [11,12]. One promising approach to making telehealth more accessible is the integration of “value-added medical education,” whereby students’ service learning improves health care [13]. Recognizing patients’ need for individualized technology support and students’ desire to serve the community, we created the Stanford Technology Access Resource Team (START) to help bridge the digital divide.

START is an undergraduate and graduate student course and volunteer program that offers immersive, meaningful patient-engaged experiences to promote patient health equity. START is a quarter-long course that teaches students about social determinants of health and health technologies and then connects them with patients with low digital technology literacy. Since digital literacy has been identified as a determinant of health, we hypothesized that this service-learning course would improve patient access to telemedicine to decrease their digital divide while also improving professional development, learner communication, and understanding of health equity and patient challenges. We conducted a comprehensive program evaluation to assess patient outcomes (connection to digital health and telemedicine and satisfaction) and learner outcomes (relationship-building skills, awareness of patient experiences, and future career life goals or purposes).

## Methods

### Ethical Considerations

The Stanford University Institutional Review Board determined this was nonhuman participant research (protocol #73006). All data have been anonymized and deidentified.

### Study Design

In the fall of 2020, amid the COVID-19 pandemic, we developed START for undergraduate and graduate students. The course addressed social determinants of health for vulnerable populations and included a service-learning component focused on digital literacy for patients in 2 health systems. The study evaluated the impact on student learning through thematic analysis of student reflections and the impact on patients through data on outreach, enrollment in EMR accounts, access to telehealth, and patient reflections.

### Study Context

Stanford University School of Medicine and Stanford Healthcare form an integrated academic medical center in Northern California, situated within an undergraduate and graduate campus. Within the surrounding counties (37 cities with large unincorporated rural areas), patients’ social determinants of health vary widely. Surrounding county median income is about US $100,000, with racial or ethnic distribution of approximately 3% African American, 30% Asian, 25% Latinx, 50% White, and 14% other, with overlap [14]. We collaborated with a federally qualified health center (FQHC) to expand our service-learning project to additional underserved community patients. The FQHC serves a population of whom 82% are living at or below 200% of the federal poverty level; 27% are uninsured; and 81% of patients identify as Latinx, African American, or Pacific Islander [15]. Stanford University encompasses 7841 undergraduate and 9688 graduate students [16]. The second most popular major for students is human biology, and approximately 10% of the campus’ student body apply to medical school each year [17,18].

### Course Development

We conducted needs assessments of students (Multimedia Appendix 1), patients (Multimedia Appendix 2), and health care teams (Multimedia Appendix 3) to inform the design of a course that would enable students to support patients who needed help accessing digital health. We distributed the student needs assessment through premed advisors, dormitory email lists, and flyers posted throughout the campus. All except 1 of the 107 student respondents agreed that the proposed course would meet a community need, and 93.5% (n=100) wished to have more information about the course. We distributed patient needs assessments to residents at 2 assisted living facilities in the community, and 125 patients completed the assessment. In total, 28% (n=35) of patients stated they would like to do telehealth visits, while 25.6% (n=32) said they had barriers to using telehealth. The top 3 reasons cited were not knowing how to connect to the telehealth platform, lack of familiarity with the internet or technology, and not having a stable internet connection. We distributed the needs assessments for health care teams to patient care coordinators at 3 of the institutional primary care clinics. In total, 6 (55%) of the 11 care coordinators recalled that more than 10 patients requested help with telehealth connection. The care coordinators cited the following barriers to providing the help: the lack of a resource line to help troubleshoot (n=8, 73%), time constraints (n=6, 55%), and the lack of training (n=5, 46%). Based on these findings, we developed a 1-unit, repeatable, 10-week-long course consisting of weekly 1-hour didactics combined with service learning to directly support patients in need of digital health support.

This course structure was chosen since the instructor wanted to balance the amount of time spent on didactics, workshops, and reflection with time reaching out to patients. The 10-week format followed the quarter system of the institution. Students are allowed to repeat the course or continue to provide service as volunteer without repeating the course. Due to the tight schedule of 10 weeks, the beginning 2 weeks of the course needed to focus on completing HIPAA (Health Insurance Portability and Accountability Act) training and training on the technical aspects of helping patients set up an EMR account and video visits. Since the course involved only telephone communication with patients, training on telephone communication skills was deemed essential. Education on the social determinants of health was key to understanding how to decrease the digital divide. Design thinking gave students a practical framework for thinking about how to solve a problem. The availability of speakers to do repeated digital sessions with the students during the COVID-19 pandemic also influenced the topics chosen.

### Didactics or Workshops

**Figure 1:**
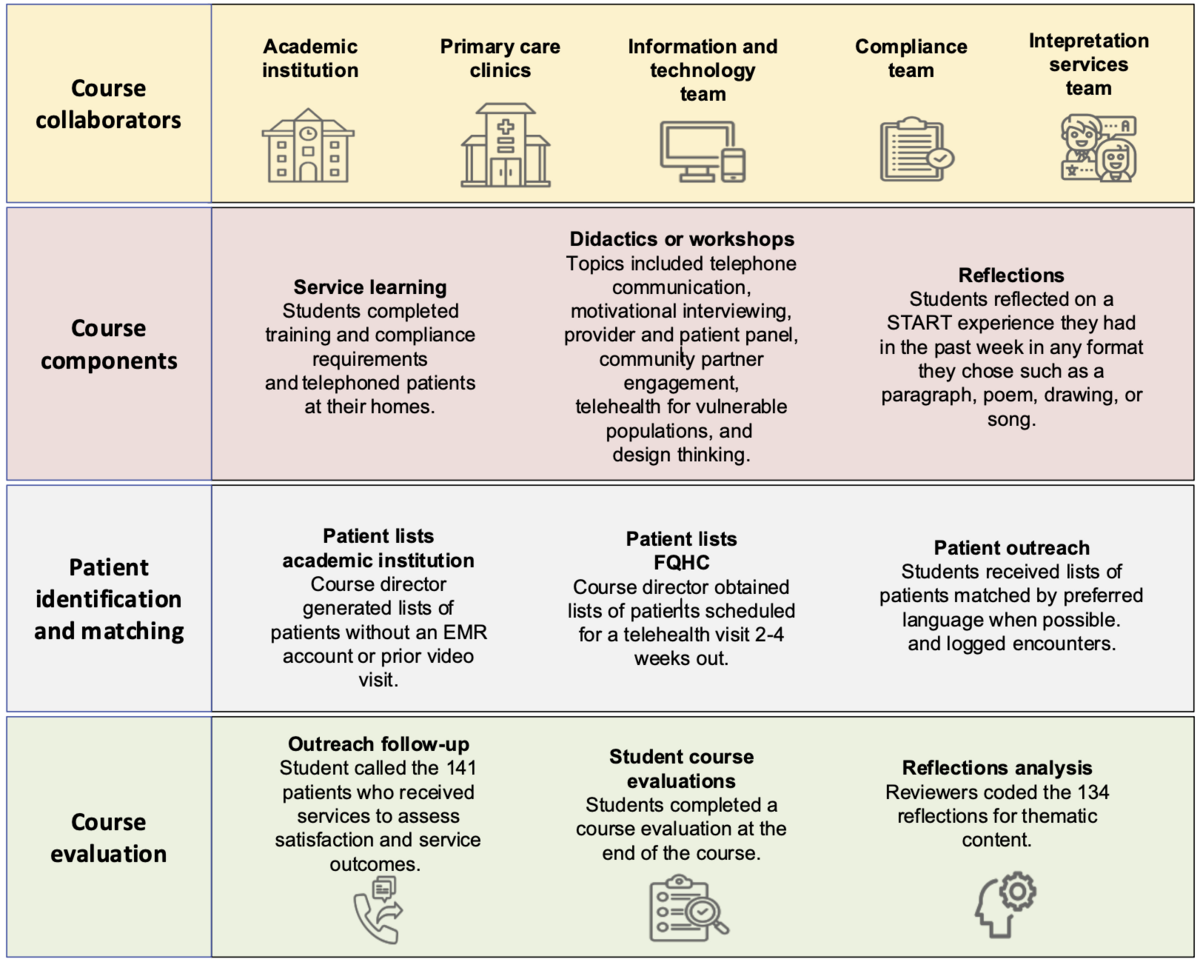
Structure of START course. EMR: electronic medical record; FQHC: federally qualified health center; START: Stanford Technology Access Resource Team.

Weekly didactics and workshops (Figure 1) focused on teaching students inclusivity when interacting with diverse patients and caregivers. Guest speakers provided insight into topics such as the telehealth experience from patient and provider perspectives, social drivers of health, motivational interviewing, and design thinking. These collaborative sessions challenged students to think critically about creating more effective solutions to address community determinants of health that impact health care access.

**Figure 2:**
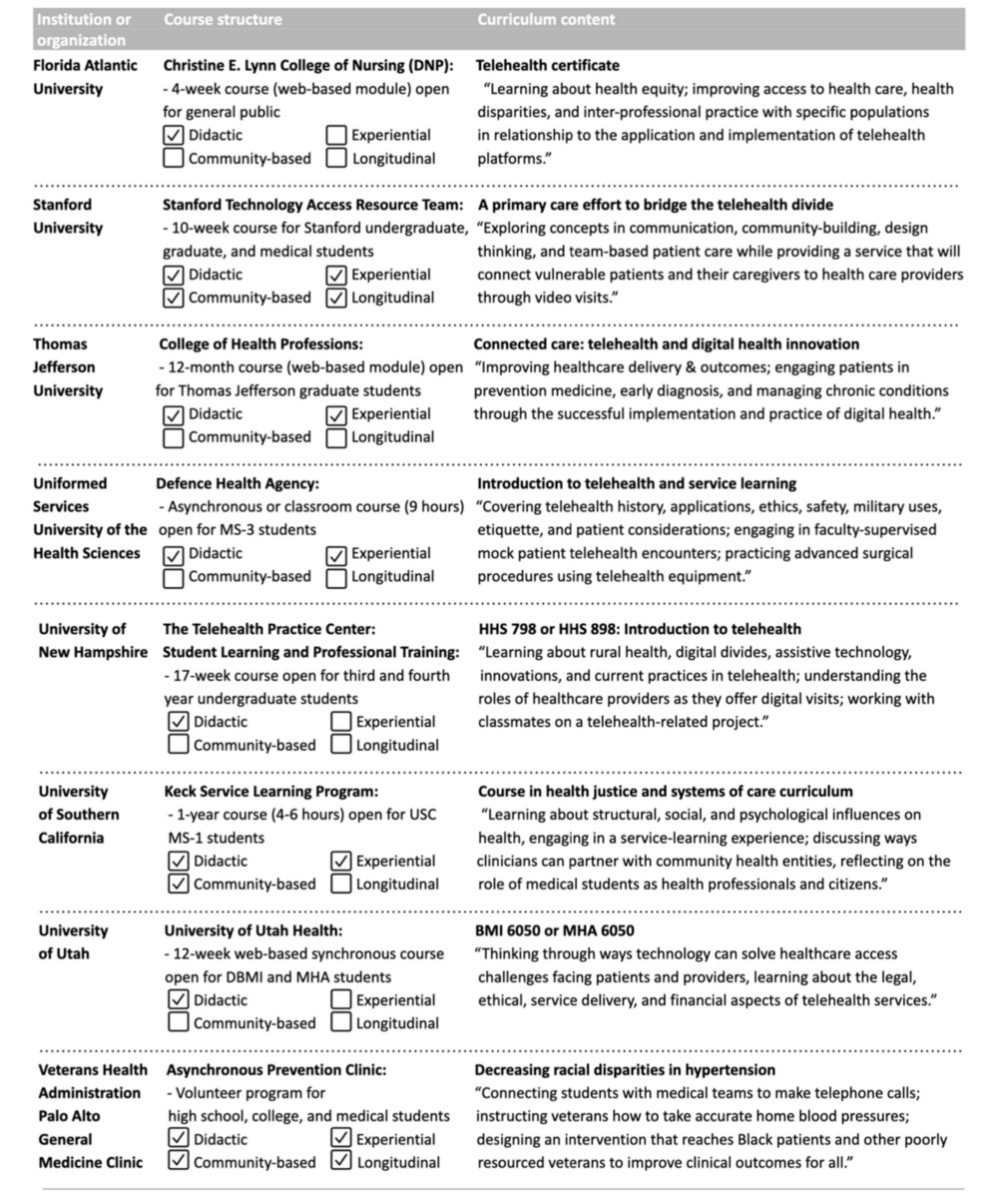
Examples of current telehealth or service-learning educational programs. DBMI: Department of Biomedical Informatics; DNP: Doctor of Nursing Practice; MHA: Master of Health Administration; MS-1: medical school year 1; MS-3: medical school year 3; USC: University of Southern California.

### Service Learning

We partnered with the institution’s informatics and technology, compliance, and interpretation services teams to develop a protocol for students to assist patients in establishing accounts in the EMR portal and video visits. The START course is open to any Stanford undergraduate or graduate student interested in service learning around medically vulnerable populations and is listed in the Stanford course directory as MED 258: Stanford Technology Access Resource Team: A Primary Care Effort to Bridge the Telehealth Divide. Once enrolled, students completed HIPAA training and other institutional compliance requirements. The institution’s informatics and technology team provided students with technical training. Students used Doximity Dialer, Cisco Jabber, and Google Voice to keep their personal phone numbers private.

We included patients from Stanford and one of our affiliated FQHCs. We identified and recruited Stanford patients for this program through EMR queries for patients seen in our primary care system in the last 2 years who lacked an EMR account or who never had a video appointment. We discussed a similar recruitment process with our FQHC partners. Many of their patients were using phone visits, and the FQHC leadership was interested in ensuring that these phone-visit patients could participate in video visits, which increased the patient-provider connection and diagnostic potential of the encounter. As such, they preferred to reach out to patients who had been seen by their primary care partner within the past 1 month and who were on the schedule for any remote clinical visit—which included both telephone and video visits within 2 to 4 weeks. We also informed the primary care clinicians of START services and encouraged referrals. After completing the necessary training, START students were each given a list of 10 patients without digital health access and called all the “remote clinical visit” patients, offering technology help to patients who did not have current video visit services. The course director prioritized language concordance between the students and patients when possible.

START students reached out to patients by telephone using scripts and resources tailored to the 2 institutions (Multimedia Appendix 4). The FQHC provided a student guidebook that included scripts and screenshots for helping patients set up EMR accounts and video visits. The institution’s technology team also developed a PowerPoint presentation to show students how to set up EMR accounts and video visits. Among the various protocols and resources, students had access to sample introductory scripts and university IT phone numbers. We have included the protocols for assistance in Multimedia Appendix 4. Following each call, START students logged each of their patient encounters using a web-based survey tool (Multimedia Appendix 5). If a patient did not pick up their phone, the student left a detailed voicemail and attempted 2 more calls. Students requested additional lists of 10 patients when ready to outreach to additional patients. Students had access to interpretation services when needed and could reach the course director outside of class time for questions or patient-specific concerns. For the nonresponders who we could not reach after multiple contact attempts, we could not draw conclusions about why they did not respond to our phone calls.

### Learner Impact

We asked students to submit weekly reflections in response to the following prompt: “Please upload a reflection of any kind - poem, art, paragraph, recording, etc. on an aspect of START you encountered this past week.” Students were given opportunities to share their reflections during class. At the end of 6 quarters, 2 independent reviewers (NC and ZND) coded the reflections for thematic content. Thematic saturation was achieved after reviewing 20 reflections. We randomly selected another 5 reflections to review to ensure no additional themes arose. During coding of the remaining 114 reflections, no new themes were identified. Two reviewers (NC and ZND) then independently assigned themes to all reflections (multiple themes could be assigned to a single reflection if applicable). A third reviewer (RB) resolved any discrepancies through consensus discussion [19,20].

### Patient Impact

Students completed patient encounter logs via a survey tool (Multimedia Appendix 5) documenting details of the call, including whether the outcome of the call was successful. In July 2023, we conducted follow-up calls with all 141 patients who expressed interest in accessing their EMR accounts a year and a half after the start of the course and video visits, and the respondents were given 4 prompts (Multimedia Appendix 6) to elicit their satisfaction with the student encounter and their success in using telehealth. We took detailed interview notes including patient quotes and conducted a thematic analysis of the responses. We did not follow-up with the 88 patients who declined support when the START student first connected with them.

## Results

### Patient Outcomes

Over 6 academic quarters (2 years), 57 students reached out to 1185 patients (n=711, 60% from an academic medical center and n=474, 40% from an FQHC). Of the 229 patients reached, 83.8% (n=192) spoke English; 10.9% (n=25) spoke Spanish; 3.9% (n=9) spoke Mandarin; and 1.3% (n=3) spoke Farsi, Korean, or Russian. Two students accessed our institution’s interpretation services. The amount of time spent on the calls ranged from less than 5 minutes to more than 60 minutes, and the majority of calls took less than 15 minutes.

Of the 229 patients reached, 141 patients desired telehealth visits but lacked access (Table 1). START students successfully established an EMR portal account and video visit setup in 78.7% (111/141) of patients who desired telehealth. The remaining 21.3% (30/141) experienced barriers to establishing access, which included lack of access to Wi-Fi or a device (n=11), absence of an interpreter (n=4), disabilities precluding the patient’s use of video visits (n=2), and unknown reasons (n=13).

**Table 1:** Outcomes from Stanford Technology Access Resource Team patient encounters (N=229).

Outcomes		Patient encounters (N=229), n (%)
**Successful outreaches**		111 (48.5)
	EMR^a^ account established	50 (21.8)
	Video visit via Doximity	36 (15.7)
	Video visit via EPIC platform	18 (7.9)
	Video visit via Zoom	7 (3.1)
**Connection but patient declined support**		88 (38.4)
	Declined assistance or did not specify	44 (19.2)
	Account already set up	23 (10)
	Preferred in-person or phone visit	18 (7.9)
	No longer in health care system	3 (1.3)
**Connection but inability to establish telemedicine access**		30 (13.1)
	Unknown	13 (5.7)
	No device or no Wi-Fi	11 (4.8)
	Interpreter not available	4 (1.7)
	Disability precluding use	2 (0.9)

^a^EMR: electronic medical record.

Over a third (88/229, 38.4%) of contacted patients did not engage with START students for the following reasons: 19.2% (44/229) declined assistance for unknown reasons, 10% (23/229) already had accounts or video visits setup, 7.9% (18/229) preferred in-person or phone appointments, and 1.3% (3/229) were no longer part of the health care system.

### START Student Outcomes

Over the course of 6 academic quarters, multiple cohorts of students submitted a total of 139 reflections. From the reflections, we identified 7 themes: real-world communication or relationship-building (n=67, 48.2%), logistics of communication (n=67, 48.2%), value of service, (n=60, 43.2%), improved awareness of patient experiences (n=38, 27.3%), patient demographics affecting telehealth access (n=35, 25.2%), future career life goal or purpose (n=19, 13.7%), and graphical or pictorial reflections (n=18, 12.9%; Table 2). On the course evaluations, 76% (39/51) of students stated they learned “a great deal” or “a lot” from the course. Future pre- and postcourse student evaluations could provide quantitative measures of student learning including any changes in communication skills and knowledge of health care systems, community-building, team-based care, and design thinking.

**Table 2:** Samples of Stanford Technology Access Resource Team (START) student reflections and illustrated themes.

Theme	Mentions (n=139), n (%)	Description	Representative quote
Real-world communication or relationship-building	67 (48.2)	Highlights the development of skills in communication or in building rapport	“Wanting to go into medicine, communicating with others is a very important skill to have. Many times, it feels like this has been lost in the age of social media, but it has been nice to have this class to sharpen my skills.” “Although the structure was scripted, I was inspired by the glimpses into the lives of the participants that they provided through their answers—from trips to the beach with a pet, to spending time in a garden.”
Logistics of communication	67 (48.2)	Documents the successes and areas for improvement regarding the START course	“This past week has been eventful as I called all my patients again that did not answer before. I finalized all my survey responses and submitted them. There were several patients who did not answer and/or had disconnected phone numbers.” “Being able to effectively communicate with people without providing them with visuals or physical assistance is often difficult.”
Value of service	60 (43.2)	Examines the student’s efforts to serve the patient or the community	“Every ring of the phone / Every click of the mouse, / Every warm “Hello,” / Every “I can’t access this page,” / Every “Oh! I see it now,” / Every appreciative “Thank you for understanding” / Every concluding “Your help is always cherished.” / Only fuels my drive, my purpose / I feel significant in the never-ending telehealth battle, / Slowly building a bridge of compassion / Within a world filled with divides.”
Improved awareness of patient experiences	38 (27.3)	Explores the student’s exposure to novel patient challenges and the effects of digital literacy on health care	“In working with patients with varied technological experience, access, and support, I have developed a strong appreciation for the privilege that we, as members of a tech-fortified community are immersed in daily, and most importantly, gained significant empathy and understanding of the challenges that many patients face in accessing essential and quality healthcare, especially technology-reliant healthcare like telemedicine.”
Patient demographics affecting telehealth access	35 (25.2)	Centers on the student’s recognition of the impact of social determinants of health on patients	“Seeing how certain industries and people responded to the pandemic shows the need for telemedicine for people who are more at risk due to age, disability, and other conditions that have made it extremely difficult for them to navigate the world.” “A recurring theme for me is how the rise of advancements in telehealth has enabled expansion of care to populations who have historically experienced high barriers to technology entry.”
Future career life goal or purpose	19 (13.7)	Focuses on the course’s impact on the student’s future career or life goal	“Med258 was a really important experience in confirming my passion to become a physician!” “Right now, I'm thinking about going into primary care—in large part because of this course.” “The most common thing I have noticed with the doctors I have shadowed is they are in and out when seeing patients. If this class has taught me anything, it is the importance of taking the time to listen to patients’ stories.”
Graphical or pictorial reflections	18 (12.9)	Illustrates START patient encounters through images and diagrams	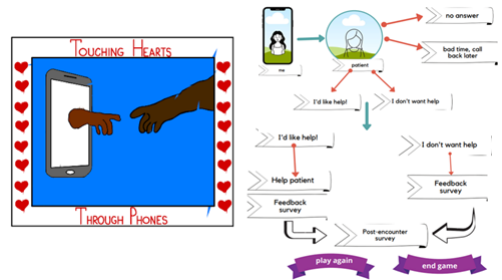

### Program Evaluation by Patients

After working with a START student for 1.5 years or more, we reviewed the records from the student logs of the encounters and the patient charts of 50 patients and found that 31 (62%) patients have had successful video visits, 8 (16%) had died, and 5 (10%) established an EMR account but did not have subsequent video visits. The status of 6 (12%) patients could not be determined due to data entry error. We successfully contacted 19 (13.5%) of the 141 patients from our primary care clinics who were helped by START students to conduct a follow-up survey. Almost all (n=18, 95%) patients reported that START students were either very (n=14, 74%) or somewhat (n=4, 21%) helpful in assisting them with health technology. The majority of patients (n=14, 74%) reported that they successfully gained access to their EMR accounts as a result of student help, while 58% (n=11) said that they connected successfully to video visits. Of the 5 patients who did not gain EMR account access, 3 patients forgot the steps and 2 patients had disabilities hindering their abilities to use the EMR or lacked access to a device.

A detailed analysis of notes regarding patient experiences, including quotes, from the follow-up calls produced 5 themes: patient satisfaction and improvement suggestions (n=19, 100%), navigation of technology logistics (n=14, 74%), activation of electronic health portal access (n=9, 47%), preparation for video visit (n=8, 42%), and support of patient scheduling (n=5, 26%; Table 3). All patients reached (n=19, 100%) expressed appreciation for student help in increasing their familiarity with the EMR. Several patients reported that students demystified the digital health experience, which included conversations about data privacy. Some patients expressed gratitude for the companionship from the student calls, highlighting the students’ “patience,” “kindness,” and “effort.”

**Table 3:** Sample patient quotes from follow-up calls and themes illustrated.

Theme (number of mentions)	Mentions (n=19), n (%)	Description	Representative quote
Patient satisfaction and improvement suggestions	19 (100)	Analyzes patient feedback on the support provided by students	“The student was very, very helpful in answering all of my questions. However, when we hung up, I forgot the steps to log into my account. I would have liked it if the student would have followed up with me again.” “The young lady was very very helpful in setting up my EMR portal account.” [When asked “How useful was your meeting with the START student in helping you connect with your doctor by EMR portal? (Scale 1-5) 5 – Very helpful, 1 – Not helpful] Patient replied: “I would rate it more than a 5, it’s a 10!”
Navigation of technology logistics	14 (74)	Explores patient struggles with telehealth technology and the assistance they receive, including step-by-step guidance from students	“When the pandemic started, I regularly used Zoom to go to church. But for some reason I couldn’t figure out how to connect with my doctor [via telehealth]. The nurses tried helping me, but I would forget the steps. So, I decided to just do voice calls with my doctor each visit ... But when the [START] student called, they walked me through the step-by-step process of how to use Zoom and was really patient with me. After a week of working with the student, I was finally able to connect with my doctor using video. The student also helped me set up my fax machine, which I was very grateful for.”
Activation of electronic health portal access	9 (47)	Examines patients gaining access to electronic health portals	“For the longest time, I wasn’t able to get an activation code set up for my [EMR portal] account. When the student called me, she was also having trouble getting my account activated ... She [the student] hung up to follow up with my primary care team. The next day, she called me back and we were able to set up my account by using my credit card number.”
Preparation for video visit	8 (42)	Focuses on the reminders patients receive from students	“I cannot fully recall the interaction with the student, but I do remember a kind young lady who called me and went through a checklist of items I should have next to me in preparation for my video visit appointment.”
Support of patient scheduling	5 (26)	Delves into the assistance provided to patients in scheduling appointments	“My mother is 94 years old ... When the pandemic was really bad, the student helped me set up a vaccine appointment for both my mom and me. Since my mom is 94, she prefers to go in person for her appointments, so we didn’t use the video visit services. However, I appreciate the student’s effort.”

## Discussion

### Principal Findings

We found that undergraduate and graduate students can function as technology navigators for patients with low digital literacy levels while gaining valuable experience in health disparities. Our students successfully guided over 100 patients through the steps necessary for digital health access, thereby connecting patients with their health care teams, supporting patients in setting up appointments, reminding patients of items to bring to their visits, and providing an empathetic perspective. Overall, patients expressed deep satisfaction with their interactions with START students, highlighting students’ positive attitudes and demystification of technology (Table 3).

Digital health literacy is an emergent social determinant of health [21]. While telehealth and technology-based tools can make health care more equitable and accessible, they can also create barriers to quality care for patients with low digital literacy [22-24]. The eHealth literacy theoretical framework by Norman and Skinner [25] explains that users of electronic health tools must be able to “seek, find, understand, and appraise” information in order to be technology literate [21]. Our study reinforces previous findings, showing patients with limited digital health literacy may successfully participate in video visits with proper education in the evolving technology landscape [21,26,27]. While asynchronous web-based tutorials may benefit many patients with technology navigation issues, others require a more personalized, hands-on approach to technology education [11,12,28]. For instance, older adults and patients with cognitive impairments require more individualized resources to increase health literacy [21]. For patients in our cohort, lack of telehealth access was more a consequence of lack of technological support or education, as opposed to lack of internet access or technology ownership.

Treating digital literacy analogously to other social determinants of health can inform both policy and health care practice. Health care providers, educators, and policy makers are well-positioned to integrate digital literacy into patient care and medical education. To address telehealth and other health technology adoption as a social determinant of health, technological support should be individualized and patient-centered [28-30]. Patient-centered digital literacy can be enhanced through interdisciplinary collaboration between technologists, public health experts, and health care providers (social work, etc) to develop new programs such as START.

In addition to positively impacting patient outcomes, integrating service learning and civic engagement in our course enhanced student learning as future health care providers [13,31-35]. Currently existing service-learning programs incorporate learner practicums on direct patient access and offer valuable opportunities for student immersion in community-based experiential work [36-39]. Building on the foundations of existing community engagement courses and programs (Table 4) [40-47], the START curriculum embeds hands-on experiences in a classroom setting to address technology literacy and access.

**Table 4:** Examples of current telehealth or service-learning educational programs.

Institution or organization	Course structure	Format	Curriculum content
Florida Atlantic University	*Christine E. Lynn College of Nursing (DNP):* 4-week course (web-based module) open for the general public	Didactic	*Telehealth certificate:* “Learning about health equity; improving access to health care, health disparities, and inter-professional practice with specific populations in relationship to the application and implementation of telehealth platforms.”
Stanford University	*Stanford Technology Access Resource Team:* 10-week course for Stanford undergraduate, graduate, and medical students	Didactic Experiential Community based Longitudinal	*A primary care effort to bridge the telehealth divide:* “Exploring concepts in communication, community-building, design thinking, and team-based patient care while providing a service that will connect vulnerable patients and their caregivers to health care providers through video visits.”
Thomas Jefferson University	*College of Health Professions:* 12-month course (web-based module) open for Thomas Jefferson graduate students	Didactic Experiential	*Connected care: telehealth and digital health innovation:* “Improving healthcare delivery & outcomes; engaging patients in prevention medicine, early diagnosis, and managing chronic conditions through the successful implementation and practice of digital health.”
Uniformed Services University of the Health Sciences	*Defence Health Agency:* Asynchronous or classroom course (9 hours) open for MS-3 students	Didactic Experiential	*Introduction to telehealth and service learning:* “Covering telehealth history, applications, ethics, safety, military uses, etiquette, and patient considerations; engaging in faculty-supervised mock patient telehealth encounters; practicing advanced surgical procedures using telehealth equipment.”
University of New Hampshire	*The Telehealth Practice Center:Student Learning and Professional Training:* 17-week course open for third- and fourth-year undergraduate students	Didactic	*HHS 798 or HHS 898: Introduction to telehealth:* “Learning about rural health, digital divides, assistive technology, innovations, and current practices in telehealth; understanding the roles of healthcare providers as they offer digital visits; working with classmates on a telehealth-related project.”
University of Southern California	*Keck Service Learning Program:* 1-year course (4-6 hours) open for USC^a^ MS-1 students	Didactic Experiential Community based	*Course in health justice and systems of care curriculum:* “Learning about structural, social, and psychological influences on health, engaging in a service-learning experience; discussing ways clinicians can partner with community health entities, reflecting on the role of medical students as health professionals and citizens.”
University of Utah	*University of Utah Health:* 12-week web-based synchronous course open for DBMI^b^ and MHA^c^ students	Didactic	*BMI 6050 or MHA 6050:* “Thinking through ways technology can solve healthcare access challenges facing patients and providers, learning about the legal, ethical, service delivery, and financial aspects of telehealth services.”
Veterans Health Administration of Palo Alto General Medicine Clinic	*Asynchronous Prevention Clinic:* Volunteer program for high school, college, and medical students	Didactic Experiential Community based Longitudinal	*Decreasing racial disparities in hypertension:* “Connecting students with medical teams to make telephone calls; instructing veterans how to take accurate home blood pressures; designing an intervention that reaches Black patients and other poorly resourced veterans to improve clinical outcomes for all.”

^a^USC: University of Southern California.

^b^DBMI: Department of Biomedical Informatics.

^c^MHA: Master of Healthcare Administration.

### Comparison to the Literature

Service-learning programs vary in terms of duration, degree of service requirements, direct versus indirect interactions with patients and caregivers, and topics covered in their curricula (Table 4) [40-47]. We found service-learning programs varying in length between a few weeks to a year, usually with a specific scientific or clinical focus, such as telehealth, equity or disparities, community health, and social determinants of health. Programs had relevant experiential components, based on their learning objectives, such as video simulations of patient encounters for video visit experiences. One service-learning program for medical students involved community-based clinical work [45].

Our START program teaches students about the upstream factors that may hinder patients’ abilities to access health care and offers students the training resources to support patients in connecting with their health care teams via telehealth. As a result, students can apply the didactic lessons they learn in classroom settings immediately to first-hand experiences working directly with patients. Given the dynamic and experiential nature of the curriculum, to our knowledge, START is currently one of the only courses or programs offered to undergraduate and graduate students that combines the best practices of service learning with direct support for patients seeking assistance in accessing their EMRs and video visits.

### Strengths and Limitations

This study has several strengths and limitations. During program development and implementation, we had access to health equity experts, EMR support, and administrative support. However, even with access to patient lists generated from EMR searches, 80.7% (n=956) of patients did not answer calls or had disconnected numbers, illustrating the need to find better ways of reaching our target patient population. When eventually connecting with patients, respondents shared that they were initially unavailable and did not recognize the phone number on the caller ID and therefore did not initially answer the call. Subsequently, calls were made through a service (Cisco Jabber) that provided the institution’s caller identification. Future efforts would benefit from appropriate caller identification, when possible, to signal to patients that the caller is an extension of the care team. Our students had the opportunity to work with patients in 2 diverse populations (an academic medical center and FQHC). While successful, student supervision and trust-building within the FQHC community took additional time and resources. This may pose a challenge for institutions without such capacities. Some communities may have additional barriers such as larger portions of patients who are not technology native. It is also worth noting that the START program is an ongoing partnership with the institution’s IT team such that the training session for the students would be updated according to changes in the institution-wide digital health platforms. Similarly, the guidebook for the FQHC site would be updated according to updates in digital health technology.

Our results are reliant on student encounter logs, which may introduce consistency bias and the Hawthorne effect in self-reported data. Given the course structure, we did not follow patients over time. Future initiatives may consider having students follow up with patients soon after their scheduled doctor appointments using the same phone numbers. Patients contacted after course may have recall bias, have forgotten specifics of the interaction, or have been influenced by social desirability bias. Subsequent assessment of interaction impact will include patient follow-up within a week regarding their interaction with START students. A total of 4.8% (n=11) of our patients were unable to get digitally connected with their health care providers due to lack of access to essential devices or Wi-Fi. Future programs may consider developing partnerships with technology providers or community organizations to send devices or Wi-Fi routers to such patients (see Multimedia Appendix 4 for examples of “Links to Local Free Wi-Fi Resources” used in this study). Students had suggested a variety of program improvements, such as collaborative debriefing during sessions and providing in-person services when possible, to mitigate the challenge of navigation solely by telephone. When technical challenges faced by patients and students arose, students connected with patients’ family members or caretakers, who were able to troubleshoot. Future initiatives should consider providing students with additional phone numbers to alternate or emergency contacts. Additionally, establishing a schedule for students to enter the clinic at designated times and obtaining direct referrals from clinicians’ primary care providers could also streamline the referral process. Finally, we recommend gathering alternative contact information from students (eg, personal emails) to track their career trajectories after completing the course or program. Future studies may also consider using pre- and postcourse student surveys assessing changes in empathy and cultural humility, which could provide quantitative and qualitative measurements of program utility on these qualities.

### Conclusions

Helping patients navigate complex health systems has called attention to an arising social determinant of health—digital literacy. Approaching the needs of patients with low digital literacy is crucial to helping them reap the benefits of high-quality and efficient health care. START is an applied, experiential, longitudinal, and community-based program that couples students’ desire for service learning with curricula on social determinants of health, health disparities, and patient-centered communication skills. Programs such as START may empower students to serve as digital health navigators and may foster more culturally humble and compassionate health care professionals. START presents a valuable opportunity for our next generation of clinicians to work with patients who are vulnerable and to develop empathy and communication skills earlier on in their academic journeys while addressing digital literacy as an emergent social determinant of health. Future service-learning programs could adapt the START model of integrating a student course with partnerships with the institution’s information and technology, compliance, and interpretation and translation services to provide services beyond connecting to video visits such as helping with applications for social services, completing advance directives for health care, navigating transportation barriers, and bridging to community partners.

## Appendix

Multimedia Appendix 1 Stanford Technology Access Resource Team student needs assessment.

Multimedia Appendix 2 Patient needs assessment.

Multimedia Appendix 3 Health care teams needs assessment.

Multimedia Appendix 4 Tips or resource sheet for Stanford Technology Access Resource Team students.

Multimedia Appendix 5 Stanford Technology Access Resource Team patient encounter log template (built into a survey app).

Multimedia Appendix 6 Follow-up survey questionnaire.

## Abbreviations

EMR: electronic medical record
FQHC: federally qualified health center
HIPAA: Health Insurance Portability and Accountability Act
START: Stanford Technology Access Resource Team
