# Supplementary material for: Bridging the Telehealth Digital Divide With Collegiate Navigators: Mixed Methods Evaluation Study of a Service-Learning Health Disparities Course
Source: JMIR Med Educ. 2024 Oct 1;10:e57077. doi: 10.2196/57077 (PMC11480730; doi:10.2196/57077)
Supplement: Multimedia Appendix 1 [file mededu_v10i1e57077_app1.docx]

**Video visits have been invaluable in bridging patients to health care during the COVID pandemic and will likely be here to stay. However, many people cannot access this valuable resource due to barriers with technology. We are working on developing a course that will engage students in helping patients attain access to video visits with their health care providers. We would like your help in designing this course and appreciate your help in answering the following questions.**

1. What is your major/course of study? Check all that apply.

☐ Life Sciences (ex. Biology, Human Biology, Chemistry, Physics)

☐ Other Sciences (ex. Engineering, CS, Earth Sciences, etc)

☐ Social Sciences/Humanities

☐ Pre-med

☐ Undeclared

☐ Other: __________________

2. What is your current level of education?

☐ Undergraduate – 1st year

☐ Undergraduate – 2nd year

☐ Undergraduate – 3rd year

☐ Undergraduate – 4th year or higher

☐ Graduate and/or Coterm

☐ Other: __________________

**This course will give students an opportunity to explore concepts in design thinking, communication, community-building, and team-based patient care while providing a service that will connect vulnerable patients and their caregivers to health care providers through video visits. It consists of weekly didactic sessions and opportunities for undergraduates and graduate students to interact with patients and health care teams by phone and video. This 1-unit online course starts with an orientation and ends with a debrief and requires a commitment of approximately 4 hours per week.**

***Please read the following course descriptions, and then rank them on a scale of 1 (strongly disagree) to 5 (strongly agree).***

This course offers a way for students to authentically contribute to improving patient care.

☐ Strongly Disagree (1) ☐ Disagree (2) ☐ Neutral (3) ☐ Agree (4) ☐ Strongly Agree (5)

This is a student service-learning opportunity in response to COVID, with credits offered.

☐ Strongly Disagree (1) ☐ Disagree (2) ☐ Neutral (3) ☐ Agree (4) ☐ Strongly Agree (5)

This course is unique. I am not aware of similar opportunities.

☐ Strongly Disagree (1) ☐ Disagree (2) ☐ Neutral (3) ☐ Agree (4) ☐ Strongly Agree (5)

I believe this course meets a need in the community.

☐ Strongly Disagree (1) ☐ Disagree (2) ☐ Neutral (3) ☐ Agree (4) ☐ Strongly Agree (5)

I feel I can teach people how to utilize video-conferencing technology with some training.

☐ Strongly Disagree (1) ☐ Disagree (2) ☐ Neutral (3) ☐ Agree (4) ☐ Strongly Agree (5)

I would enjoy interacting with people from different backgrounds with different levels of experience.

☐ Strongly Disagree (1) ☐ Disagree (2) ☐ Neutral (3) ☐ Agree (4) ☐ Strongly Agree (5)

***Who are you most interested in interacting with throughout this course? Please rank the following with 1 being the group you are least interested in interacting with.***

Patients & caregivers

☐ Strongly Disagree (1) ☐ Disagree (2) ☐ Neutral (3) ☐ Agree (4) ☐ Strongly Agree (5)

Team members in medical clinics

☐ Strongly Disagree (1) ☐ Disagree (2) ☐ Neutral (3) ☐ Agree (4) ☐ Strongly Agree (5)

Hospital administrators

☐ Strongly Disagree (1) ☐ Disagree (2) ☐ Neutral (3) ☐ Agree (4) ☐ Strongly Agree (5)

Hospital tech

☐ Strongly Disagree (1) ☐ Disagree (2) ☐ Neutral (3) ☐ Agree (4) ☐ Strongly Agree (5)

Other students

☐ Strongly Disagree (1) ☐ Disagree (2) ☐ Neutral (3) ☐ Agree (4) ☐ Strongly Agree (5)

***If you were to take this course, which quarter would be most suitable for you?***

☐ Autumn

☐ Winter

☐ Spring

☐ Summer

***Which day of the week would be the best day for the didactic session? Please rank the following with 1 being the best day.***

Monday: ☐ 1 ☐ 2 ☐ 3 ☐ 4 ☐ 5

Tuesday: ☐ 1 ☐ 2 ☐ 3 ☐ 4 ☐ 5

Wednesday: ☐ 1 ☐ 2 ☐ 3 ☐ 4 ☐ 5

Thursday: ☐ 1 ☐ 2 ☐ 3 ☐ 4 ☐ 5

Friday: ☐ 1 ☐ 2 ☐ 3 ☐ 4 ☐ 5

***What would be your preferred time in Pacific Standard Time (PST) for the didactic session? Please rank the following with 1 being the best time.***

☐ 8:30AM-9:30AM PST

☐ 11:30AM-12:30PM PST

☐ 1:00PM-2:00PM PST

☐ 5:30PM-6:30PM PST

***What languages, other than English, do you speak fluently?***

__________________

***Would you be interested in learning to work with medical interpreters to communicate with patients and/or caregivers who speak a language different than you?***

☐ Yes

☐ No

☐ Maybe

***Thanks for participating and for your passion and honesty. The content of this survey will be used to design the course. Your responses will remain confidential and anonymous. However, if you would like to be contacted with the final information on the course, you may provide your name and contact information below!***

Name

__________________

Phone Number/Email/Other Contact Info

__________________

Any other comments you wish to share? (optional)

__________________
