# Supplementary material for: Bridging the Telehealth Digital Divide With Collegiate Navigators: Mixed Methods Evaluation Study of a Service-Learning Health Disparities Course
Source: JMIR Med Educ. 2024 Oct 1;10:e57077. doi: 10.2196/57077 (PMC11480730; doi:10.2196/57077)
Supplement: Multimedia Appendix 2 [file mededu_v10i1e57077_app2.docx]

**Telehealth Patient Survey**

1. Are you a (check one): ☐ Patient ☐ Caregiver/proxy answering on behalf of the patient

2. What is your (the patient’s) gender? ☐ Male ☐ Female

3. What is your age? _______

4. What is your preferred language? ☐ English ☐ Spanish ☐ Mandarin ☐ Cantonese ☐ Tagalog ☐ Vietnamese ☐ Korean ☐ Russian ☐ Other: ____

5. What is your highest level of education?

☐ 12^th^ grade or less

☐ Graduated high school or equivalent

☐ Some college, no degree

☐ Associate degree

☐ Bachelor’s degree

☐ Post-graduate degree

6. What is your living situation? Please check all that apply:

☐ I live by myself

☐ I live with other family member(s)

☐ I live with housemates/roommates

☐ I live in Lytton Gardens Senior Community

☐ I live in the Sequoias-Portola Valley

☐ I live in a different assisted living facility: ______________

☐ Other:__________________

7. Where do you get your primary care mainly?

☐ Stanford ☐ Kaiser ☐ Sutter Health (PAMF) ☐ Other: _________

8. What is your race/ethnicity? Please mark the one box that describes the race/ethnicity category with which you primarily identify:

☐ Hispanic or Latino

☐ White

☐ Black or African American

☐ Asian

☐ Native Hawaiian or Other Pacific Islander

☐ American Indian or Alaska Native

☐ Two or More Races

☐ I choose not to self-identify my race/ethnicity at this time.

**The next few questions ask about your (the resident’s) experience with devices like a phone, smartphone (iPhone or Android), or computer.**

1. Preferred device for remote visits with your clinician or healthcare team:

☐ Regular phone (not smartphone)

☐ Smartphone

☐ iPad/Tablet

☐ Computer

☐ I don’t have any of the above

2. I feel comfortable with smart devices like smartphones, iPads, or the computer:

☐ Strongly Disagree ☐ Disagree ☐ Neither ☐ Agree ☐ Strongly Agree

3. I know how to get online and use the internet without issues:

☐ Strongly Disagree ☐ Disagree ☐ Neither ☐ Agree ☐ Strongly Agree

4. I know how to connect with my healthcare team through video visits:

☐ Strongly Disagree ☐ Disagree ☐ Neither ☐ Agree ☐ Strongly Agree

5. I know how to connect with my healthcare team through telephone:

☐ Strongly Disagree ☐ Disagree ☐ Neither ☐ Agree ☐ Strongly Agree

**The next few questions ask about how you would like to connect with your care team and if you need help with the technology.**

1. I would like to be able to connect with my healthcare team through video visits:

☐ Strongly Disagree ☐ Disagree ☐ Neither ☐ Agree ☐ Strongly Agree

2. I would like to be able to connect with my healthcare team through telephone visits: ☐ Strongly Disagree ☐ Disagree ☐ Neither ☐ Agree ☐ Strongly Agree

3. I have someone who can help me access video visits if I have trouble:

☐ Strongly Disagree ☐ Disagree ☐ Neither ☐ Agree ☐ Strongly Agree

4. Learning to get better at using technology is worthwhile for me:

☐ Strongly Disagree ☐ Disagree ☐ Neither ☐ Agree ☐ Strongly Agree

5. Biggest barriers connecting with my care team through video visits are (check up to 3): ☐ Difficulty hearing well enough to participate

☐ Difficulty seeing well enough to interact with the screen

☐ Problems speaking or making oneself understood

☐ Problems with attention or memory

☐ Not having stable internet connection

☐ Not familiar with how to use the technology or internet

☐ Not knowing how to get connected to the platform

☐ No smart device (iPad, iPhone computer) available

☐ Cannot speak English very well

☐ Not interested in seeing provider outside of the clinic

☐ No perceived barriers

☐Other: ________________

6. I am interested in having more training or having someone reach out to help me better connect through video visits with my healthcare team:

☐ Strongly Disagree ☐ Disagree ☐ Neither ☐ Agree ☐ Strongly Agree

7. Any other comments? ________________

-----OPTIONAL QUESTION/DETACH HERE----

Would you be open to having a student call you to help you set-up for a video visit? If yes, please complete the following:

Name: ________________

Best phone number to contact you: ________________

Best times to contact you: ________________
