# Supplementary material for: Bridging the Telehealth Digital Divide With Collegiate Navigators: Mixed Methods Evaluation Study of a Service-Learning Health Disparities Course
Source: JMIR Med Educ. 2024 Oct 1;10:e57077. doi: 10.2196/57077 (PMC11480730; doi:10.2196/57077)
Supplement: Multimedia Appendix 3 [file mededu_v10i1e57077_app3.docx]

**Stanford Technology Access Resource Team Patient Care Coordinator Survey**

1. About how many patients have you come across with difficulty accessing video visits? **_______**

2. Have you ever assisted patients with set-up for a video visit? ☐ Yes ☐ No If yes, about how many patients have you helped? If no, skip to question 6

3. About what percentage of the time would you say you have been successful in helping patients set up for a video visit? ☐ 100% ☐ 75% ☐ 50% ☐ 25% ☐ 0

4. The most frustrating part about helping patients set up for video visits is: ____________

5. What is your feeling about helping patients set up for video visits? Please check all that apply: ☐ I enjoy helping patients in this way

☐ I usually have time to help patients with the set-up

☐ I wish I didn’t have to do it

☐ I feel I have a good understanding of how to help patients set up for video visits

☐ I know who to call if I cannot help patients troubleshoot issues with video visit set-up

☐ I received training on how to set-up video visits

☐ Comments: _____________

6. Would you be open to communicating with students who want to help patients set-up for video visits? ☐ Yes ☐ No ☐ Comments: _____________

7. If you wanted to refer a patient for help with video visit set-up, what method of referral would you prefer?

☐ EPIC message

☐ EPIC referral

☐ Email

☐ Other: _____________

☐ Comments: _____________

8. Do you have any suggestions for helping patients with difficulty accessing video visits? ☐ Comments: _____________
