# Supplementary figures and images for: Bridging the Telehealth Digital Divide With Collegiate Navigators: Mixed Methods Evaluation Study of a Service-Learning Health Disparities Course
Source: JMIR Med Educ. 2024 Oct 1;10:e57077. doi: 10.2196/57077 (PMC11480730; doi:10.2196/57077)

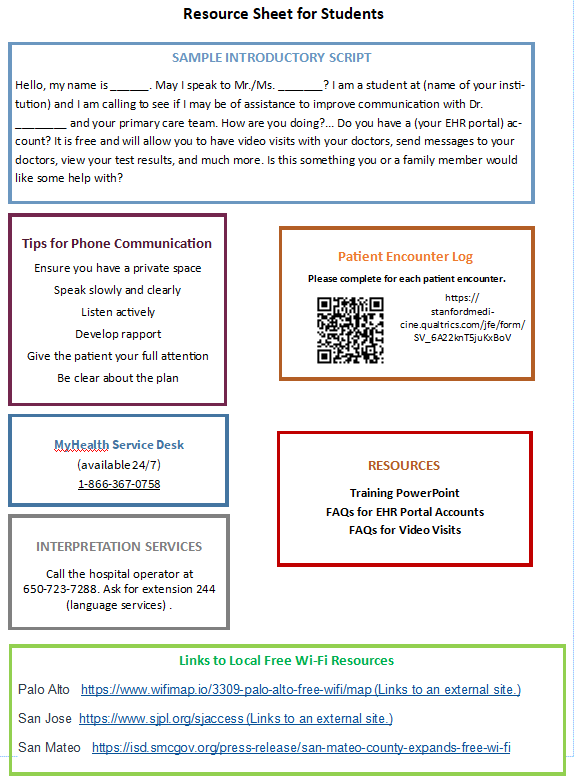

Supplement: Multimedia Appendix 4 [file mededu_v10i1e57077_app4.docx]
