# Supplementary material for: Bridging the Telehealth Digital Divide With Collegiate Navigators: Mixed Methods Evaluation Study of a Service-Learning Health Disparities Course
Source: JMIR Med Educ. 2024 Oct 1;10:e57077. doi: 10.2196/57077 (PMC11480730; doi:10.2196/57077)
Supplement: Multimedia Appendix 5 [file mededu_v10i1e57077_app5.docx]

1. Your name: _____________

2. Please enter the patient's initials, please include middle initials: _____________

3. The patient was part of this health care system (if students are working with patients from different health care systems): _____________

4. Please select all answers that apply. The encounter involved working with:

☐ The patient

☐ A care provider

☐ The patient declined help

☐ Please add any comments below: _____________

5. The patient/caregiver contacted was:

☐ From a patient list

☐ Referred by one of the providers

☐ Other

☐ Comments: _____________

6. Please add any additional notes about your encounter with either the patient or care provider: _____________

7. The encounter was conducted in:

☐ English

☐ Spanish

☐ Mandarin/Cantonese

☐ Other. Please specify which language: _____________

8. An interpreter was used: ☐ Yes ☐ No

7. Please select all that apply. I was able to help the patient set up a:

☐ EMR portal account

☐ Video visit through the EMR platform (MyChart/EMR portal)

☐ Video visit via Doximity

☐ Video visit via Zoom

☐ Other

8. The number of minutes I spent on this patient encounter are (total if you needed to make more than one call):

☐ 0-5

☐ 6-15

☐ 16-30

☐ 30-60

☐ >60

9. The number of calls made to complete service to patient:

☐ 1

☐ 2

☐ 3

☐ >3: Please specify number: ____

10. Patient stated that they would like additional support with:

☐ Additional technical support for video visits

☐ Additional technical support in EMR portal or MyChart

☐ Clinical Questions

☐ Other (please provide more information): _____________

☐ Additional Comments or Observations: _____________
