# Supplementary material for: Bridging the Telehealth Digital Divide With Collegiate Navigators: Mixed Methods Evaluation Study of a Service-Learning Health Disparities Course
Source: JMIR Med Educ. 2024 Oct 1;10:e57077. doi: 10.2196/57077 (PMC11480730; doi:10.2196/57077)
Supplement: Multimedia Appendix 6 [file mededu_v10i1e57077_app6.docx]

1. How useful was your meeting with the START student in helping you connect with your doctor via EMR portal? (Scale 1-5) 1 – Not helpful, 5 – Very helpful

2) Did the student help you answer questions with regard to preparing for your video visit and how? (Other general questions relating to non-technology issues (e.g. medications, blood pressure, etc.)

3) Did you have any technology problems when you had your video appointment/were you able to connect? How was your video appointment?

4) Was there anything you wish you had been told about during your video visit? How else can we support you to have a great video visit?
